# A big data association rule mining based approach for energy building behaviour analysis in an IoT environment

**DOI:** 10.1038/s41598-023-47056-1

**Published:** 2023-11-13

**Authors:** M. Dolores, Carlos Fernandez-Basso, Juan Gómez-Romero, Maria J. Martin-Bautista

**Affiliations:** 1 Department of Computer Science and Artificial Intelligence, University of Granada, Periodista Rafael Gómez Montero 2, 18015 Granada, Andalusia Spain; 2 Department of Experimental Psychology, University College London, 26 Bedford Way, London, WC1H 0AP UK

**Keywords:** Computer science, Computational science, Information technology, Scientific data

## Abstract

The enormous amount of data generated by sensors and other data sources in modern grid management systems requires new infrastructures, such as IoT (Internet of Things) and Big Data architectures. This, in combination with Data Mining techniques, allows the management and processing of all these heterogeneous massive data in order to discover new insights that can help to reduce the energy consumption of the building. In this paper, we describe a developed methodology for an Internet of Things (IoT) system based on a robust big data architecture. This innovative approach, combined with the power of Spark algorithms, has been proven to uncover rules representing hidden connections and patterns in the data extracted from a building in Bucharest. These uncovered patterns were essential for improving the building’s energy efficiency.

## Introduction

Every single minute a modern building management system can generate thousands of readings from different sensors such as temperature, air conditioning, thermostats, window open/close sensors, pressure sensors, etc. that register its operation^1^. Companies in the Energy field are every day more conscious of the great opportunity that the analysis of these data can bring to them. As a consequence, there is a tendency of increasing the inclusion of smart meters that monitor every corner of the building, generating, when fully deployed, more than 1000 petabytes of data annually^2^. To handle all these data and be able to analyse them, new Big Data technologies can be a good ally. Around this concept of Big Data, there are several frameworks and technologies that help to organize, store and transform the data into valuable information for the building owners and/or power companies which provides the energy. Since the data collected in the architecture involves massive data sources, it is necessary to apply new distributed algorithms that can handle, process and extract new information from large amounts of data. The main challenge in the field of energy Big Data is therefore to provide adequate techniques to help analysts understand which of the available data is useful to answer the questions they are interested in and relevant for decision making. These questions focus on understanding the context of energy systems, exploring and profiling the data and applying statistical and machine learning methods are essential steps. Other applications such as feature engineering and data governance further contribute to data selection, while iterative analysis and data visualisation help to understand the results and to extract hidden knowledge from the results^3^.

Although Big Data presents new opportunities^4–6^, it is also arduous to store and handle such huge and complex datasets containing very heterogeneous data that are very different from the constitution, and even those that could be similar are collected in different time instants or do not have the same granularity. But gathering, storing and analysing the data are not the whole story. One intrinsic limitation of some supervised algorithms is the lack of reliable training data that can be used to predict the energy-building behaviour. By contrast, unsupervised techniques do not have that restriction, focusing on the discovery of the hidden correlations and associations in the data, thus requiring less domain expertise.

**Figure 1:**
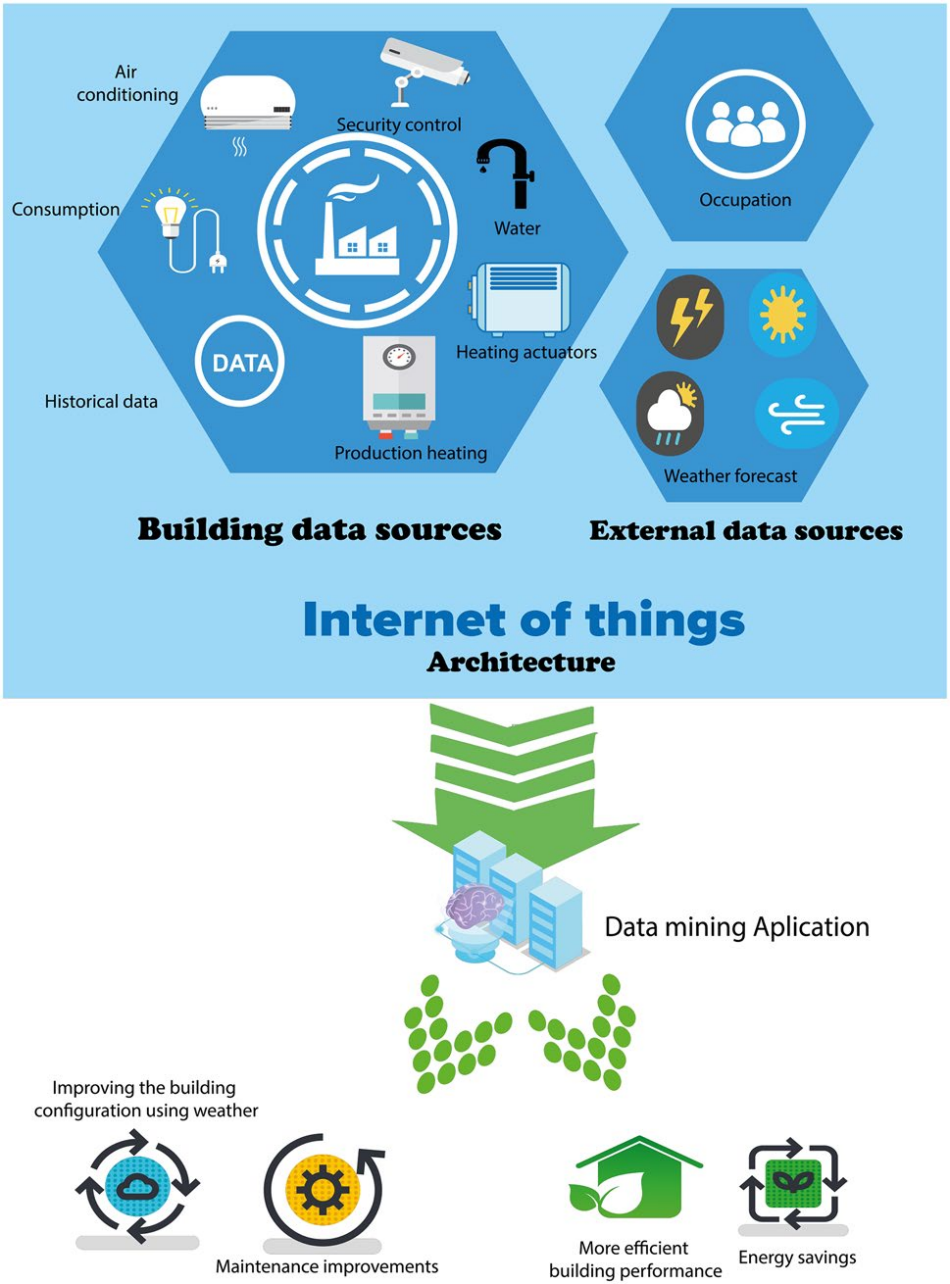
IoT architecture.

The main contributions of this work are:

- We propose an IoT data architecture (see Fig. 1) using a Big Data framework whose core is based on association rules discovery^7^, an unsupervised technique capable of finding relationships between variables and their values. For that, we have employed the Spark platform^1,8,9^ based on the Map-Reduce paradigm that enables a distributed computation of big data volumes. This platform following the NoSQL specifications, will help to store the produced building data with a dynamic schema, something that is extremely useful when data have to be used by different programs and for different purposes, not only by knowledge discovery processes.
- The methodology proposed is oriented towards the reduction of energy use in buildings. In this paper, the experiments using this methodology have been carried out in different time periods (10 days, 1 month and 3 months) in data coming from an office building located in Bucharest (Romania) obtaining some energy patterns that help to understand the overall functioning of the building for energy reduction consumption purposes.
- The obtained patterns help to discover some insights about the functioning of the building and its interaction with its occupants in order to improve the energy efficiency of the building.

The work is structured as follows. Next section reviews previous related works and introduces the necessary background of related concepts. In Section "IoT architecture using a Big Data mining methodology" it is explained how the IoT architecture works to obtain the data and the application of data mining to extract the hidden patterns. Section "Case study" shows a real use case for the application of this methodology and architecture in an office building located in Romania. Finally, in Section "Conclusions and future improvements" we summarize the conclusions and present future prospective research lines.

## Association rule mining in the field of energy building management using a IoT architecture

IoT architectures are ideal for the acquisition and management of data from large buildings^10–12^. The studies discuss the application of a Smart City Model to a traditional university campus using IoT and big data, aiming to improve sustainability, resource management, and urban comfort. This is because these buildings have a wide variety of data sources, of different types, which need to be merged, stored and managed in an efficient and coordinated approach. These systems have already been used in some applications^13–15^. In these studies, unsupervised techniques such as clustering and predictive algorithms have been used to help predict consumption and support reactive control systems. In general, these techniques focus on identifying patterns and trends in energy consumption.

This type of solution allows the storage of data from different sources. For instance, in the case of our application, data were collected from different sources including the operational data from heating and air conditioning systems, heating production systems, energy^16^, security systems^17^. These include heterogeneous data such as sensors, agendas that allow us to know the occupation, the weather forecast or data from security devices. All this amount of data must be stored correctly in each of the systems. The proposed architecture manages all these sources, supplying also the necessary mechanisms to select and merge data to enable a posterior analysis employing data mining techniques in Big Data.

In Fig. 1 we can see a complete schema of our proposal, in which we can see in the upper part the different data sources that we can find in a building and that we will detail in the use case. As it can be seen, the system is capable of managing heterogeneous data sources in the building itself that generate large amounts of data such as temperature sensors, heating or air conditioning machinery sensors, electricity or security. In addition, the architecture adds external sources such as the building’s diaries, which give information about the occupancy of the building and the weather forecast, which are very important elements when heating or cooling of the building want to be kept at a comfortable temperature for its workers.

All these data are stored using NoSQL databases and queuing systems for its correct operation and storage. In particular, we employ a MongoDB system using its *replica set* features on a Docker container system^18^. This allows the system to have a large data collection capacity even when receiving data at high frequency. From this architecture we can filter and select the data to which we apply our data mining algorithms.

In the next step, in order to extract hidden knowledge from the generated data, the system applies Data Mining techniques. These techniques have been employed within the Energy field in several areas in Refs.^19,20^ or Refs.^21–23^. In the former, it is reviewed how some of the traditional Data Mining techniques have been used to analyse building-related data^19^. In the review offered in Yu et al.^20^ the authors analysed the main challenges of the Data Mining techniques in the energy field undertaking predictive or descriptive tasks. In Molina-Solana et al.^21^, the authors focused on the different Data Mining techniques employed for Energy Management, especially in the building sector, discussing the main challenges and opportunities that will arise with the advent of new computational technologies such as Big Data. A more recent review can be found in Fan et al.^22^ oriented to analyse unsupervised data mining techniques taking special attention to mining massive building operational data. Among these techniques, association rule mining stands out as a suitable tool to discover significant patterns in operational data.

Association rules have been employed to discover meaningful and easy to interpret information^7^ that can be utilised for different scopes within the energy management field. We describe the most significant works in the following.

Association rules can be applied directly to find and discover associations in building operation data like in Refs.^24–29^, or in combination with other techniques. In particular, association rules have been employed to establish relations among occupancy and energy waste, as for instance in Refs.^25,26^. In both works the association rules helped to understand the waste of energy due to lighting by discovering relationships among time, occupancy and lighting-related energy waste in classrooms and an University library respectively. The proposal described in^27^ was comprised of several steps including data cleaning and discretization into categories representing three levels (“low”, “medium”, and “high”) according to the original numerical magnitudes. Subsequently, an association rule discovering process was applied to find potential knowledge that deviated from the domain knowledge and served to identify different regulation strategies and energy inefficient operations. The procedure was applied to collected data from two district heaters in China.

In Fernandez-Basso et al.^30^ it was proposed a general Data Mining framework, which includes among its techniques the association rule discovery tool that was employed to examine correlations among building operational data in two different time periods: a day and a year. This procedure was able to identify the energy waste in the air-conditioning system, detect some equipment faults in two fan units and propose low-cost strategies for saving energy in the building operation.

Very often, association rules have been applied in combination with clustering techniques, like the approach proposed in Xiao and Fan^31^ to unveil the relations among power consumption of major components in each cluster. An interesting proposal is also described in Wijayasekara et al.^32^ about a combined strategy using linguistic descriptions and fuzzy logic rule extraction, via a clustering algorithm, to detect possible anomalies in the regular building functioning. The work presented in D’Oca and Hong^33^, also combined clustering with association rule mining to find meaningful window opening and closing behaviour patterns in order to design recommendations for natural ventilation. This enabled to improve the thermal comfort and productivity in the office buildings. A generic framework for knowledge discovery was presented in Fan et al.^34^, which combines ANOVA variance analysis to identify most significant variables, and clustering to find the best data division according to the significant variables for, in a further step, obtaining quantitative association rules in the different data clusters. The discovered rules helped to identify some changes in building operating strategies, as well as some fault detection in power consumption sensors. In Ashouri et al.^35^ a saving recommendation system was developed and applied to eight individual houses in six different locations in Japan. For that, a detailed energy consumption of the household appliances and weather data were collected. The system independently mined association rules from the different identified clusters and afterwords, the obtained set of rules, were in turn, classified into recommendations, savings or neutral rules, in order to give useful information to users about their energy consumption.

The combination of association rules with other techniques different than clustering also appears as a good option. For instance, in Guerrero et al.^36^ León et al. implemented a proposal to detect Non-Technical Loses (NTLs) and recover electrical energy (lost by abnormalities or fraud). For that, they propose a predictive analysis tool combining a tree classification method with association rules discovery. The same authors proposed in^37^ an expert system for NTLs detection to study the customers’ consumption trends, complementing the obtained results by regression analysis with text mining techniques applied to the inspector commentaries, and association rule mining to customers data from the electric company. The work in Fan and Xiao^38^ proposes a Data Mining procedure to analyse massive operational data. The procedure relies in a model of the data partitioning based on a decision tree which gives a clue on how the data is partitioned. As an example, the time variables were divided into peak and non-peak hours taking into account if the day was a labour day or a day corresponding to holidays or weekends. Once the data was pre-processed, a genetic algorithm for quantitative association rule mining, called QuantMiner, was applied but restricted to only one antecedent and one consequent in the body of the rules to obtain more interpretative results. The obtained set of associations, in data from a university building in Hong Kong, unveiled some energy conservation opportunities in pumps, chillers and cooling tower operations.

Moreover, different association rule mining techniques, e.g. temporal association rules or gradual rules have been applied within the energy field. For instance, temporal association rule mining was applied in Fan et al.^39^ to identify associations between the operations in different subsystems taking into account a temporal dimension, with different levels of granularity, which is always included in the rule. In Fan and Xiao^40^ and Fan et al.^41^, the same authors proposed a similar Data Mining procedure but using gradual patterns in the form of association rules that indicate tendencies of the form “*the more/less A, the more/less B*” indicating operational patterns that are used, in a further step, to construct a knowledge base for detecting abnormal operating conditions in Fan and Xiao^40^ and for identifying energy conservation opportunities in Fan et al.^41^.

A completely different study was made in Thomas and Lieutenant^42^ about the projects that have been certified in Leadership in Energy and Environmental Design for new construction using association rule mining to analyse them. In particular, some interesting results were found that revealed the interplay of credit bundles in sustainable design strategies.

The core of our proposal is based on association rule discovery to discover meaningful patterns relating to energy consumption and other factors in an office building in Romania. The system starts from the data collection, including the data preprocessing, the application of association rules and different types of visualization that help to the inspection of obtained results. The proposed system takes benefit of the Big Data framework to provide the capability of storing and analysing big amounts of data in a faster way than traditional algorithms.

## IoT architecture using a Big Data mining methodology

In this work, we proposed a methodology capable of identifying potentially useful and understandable patterns of power consumption using the values from different suppliers that collect data in an energy building management system, weather forecast, occupancy and other variables stored in an IoT architecture.

The requirements needed to deploy this type of methodology are at least a set of sensors measuring the performance of the building like HVAC sensors. Additionally, other information of interest such as the agenda of occupancy of the building, and/or the weather forecast would be of consideration for a better building monitorization. Regarding the computing infrastructure, a server to run the software and store the data would be needed although it is not necessary that it is placed in the same building. Alternatives like cloud computing and remote processing might be considered. Several servers were used for the entire implementation. One of them was used to manage the databases, sensors and storage of results, with the following specifications: 16 cores, 8 TB of memory and 120 GB of RAM. On the other hand, the execution of the algorithms in Big Data was carried out on a cluster equipped with 32 cores using Intel Xeon E5 processors and 200 GB of RAM. All servers were located off-site.

For the development of this methodology, we employed an association rule mining algorithm implemented in Spark, a Big Data framework that enables a fast processing of massive amounts of data. This framework is based on the MapReduce paradigm. It was developed by Google in 2003. Since then, there have been several proposals for processing data in Big Data, with some variations. Two different frameworks have emerged as the most common when it comes to implementing algorithms in MapReduce: Hadoop^43^ and Spark^44^. These platforms have been improved in recent years by incorporating various features to make the most of a cluster’s processing capacity, allowing more scalable algorithms and significantly improving traditional forms of cluster programming. The choice of Spark is based on the fact that its in-memory processing accelerates computation. This can improve the efficiency of algorithms up to 100 times^45–47^ compared to a sequential algorithm and 10 times compared to versions using Hadoop. The extent of this improvement depends on the specific data cluster used and the distribution of the process. Non-distributable components or excessive network usage can potentially have adverse effects on the integrity and performance of this data processing process^48–50^. In our case, the process makes minimal use of the network except in the frequent itemset extraction process.

Before designing the whole methodology we took into account some important factors:

- The Big Data framework offers a new perspective for storing and processing large amounts of data, enabling the management and processing of a wide variety of data.
- MapReduce paradigm offers a fault tolerance system that ensures the continuous running of the algorithms in case the access to the data fails, etc.
- The implementation of mining algorithms using Spark enables the processing of massive data, whilst traditional algorithms fail.
- The results should be stored in a convenient way for a later visualization to enhance the comprehension of results.

**Figure 2:**
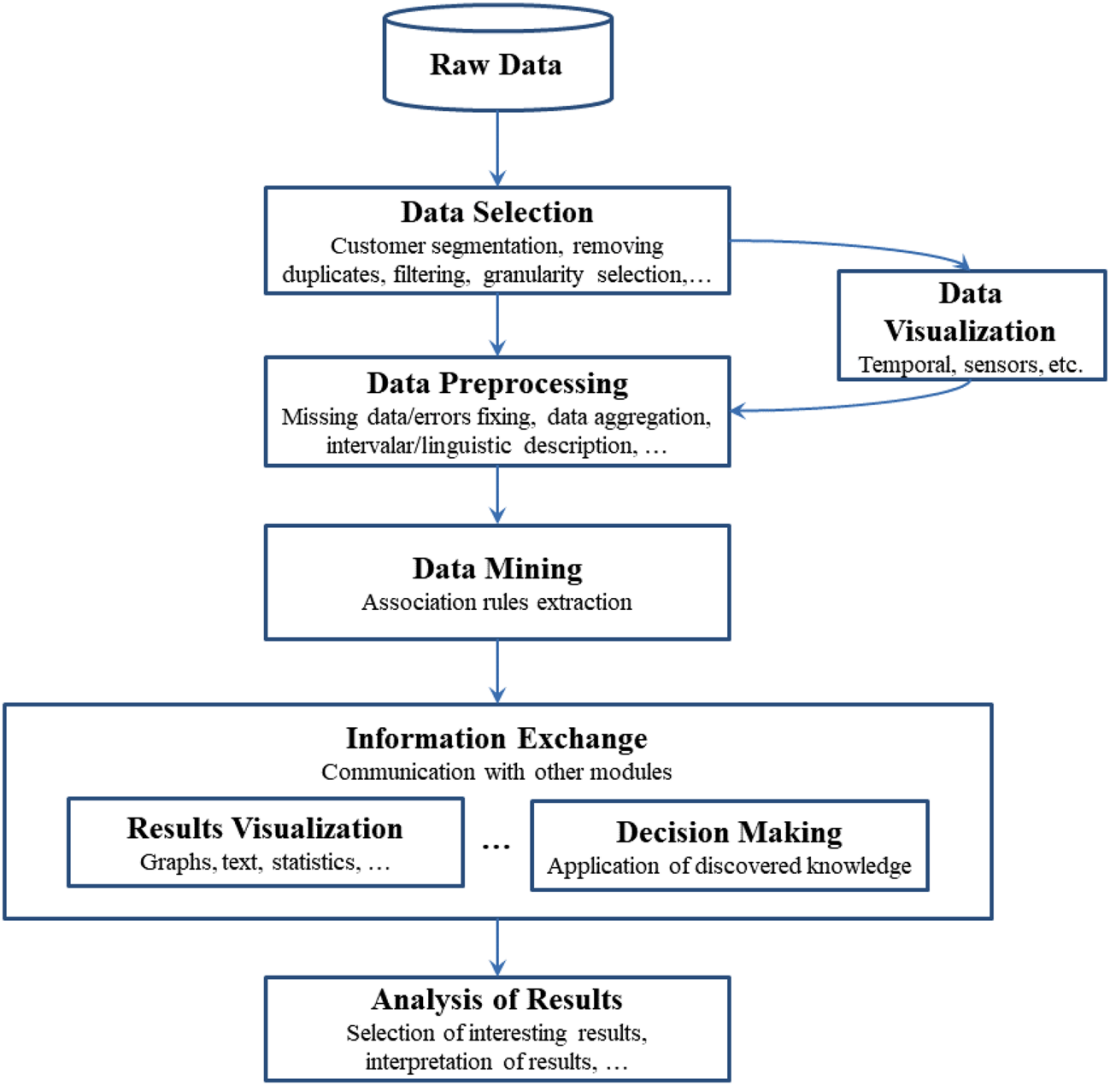
General workflow of the proposed Data mining framework.

For these reasons, a storage solution following the NoSQL specifications has been employed in this work. Several types of NoSQL databases can be chosen^51^ like for instance MongoDB, CouchDB or ArangoDB. This choice should be based on the specific requirements of the project. MongoDB is known for its flexibility and scalability, CouchDB emphasizes eventual consistency and simplicity, and ArangoDB excels in handling multiple data models, including graph data. Each database has its own strengths. In our case, MongoDB has been used for storing the filtered and preprocessed data as well as the results obtained by the Data Mining algorithm. MongoDB^52^ enables to store a data structure with a dynamic schema similar to JSON. This is extremely useful when data have to be used by different programs and for different purposes. Additionally, NoSQL specification enables a fast retrieval of results by imposing different criteria according to the fields of the database schema. For instance, we may want to retrieve all association rules obtained using data from a specific time period; or rank the obtained associations with a support higher than a specified value; or retrieve only those associations that contain in the antecedent a particular sensor; etc. For the information exchange among different modules of the Building Energy Management System (BEMS) the JSON (JavaScript Object Notation) format was employed facilitating, for instance, the retrieval of results for their later visualization or for their use in the decision making module (see Fig. 2).

The following sections explain in more detail each of the steps of the process.

### Data selection and preprocessing from IoT architecture

Continuous streams of raw data are generated by an array of meters and sensors distributed throughout the building. This deluge of information is effectively harnessed and managed by the Building Management System (BMS), which plays a pivotal role in handling this massive data influx. Serving as the central nexus, the BMS not only collects but also efficiently stores and organizes the data stemming from the myriad sensors and meters integrated within the building’s infrastructure. This dynamic synergy of big data and real-time streaming data empowers the BMS to optimize building operations with precision and agility.

The collected data sets are truly diverse, stemming from a wide array of sources within the building’s ecosystem. These sources include electrical meters, temperature sensors, humidity sensors, heating and cooling systems, water meters, and more. The sheer diversity of these data sources means that the information they provide can vary significantly in format, structure, and content.

In order to harness the full potential of this data for any meaningful analysis, a preliminary stage of preprocessing is necessary. This preprocessing step involves cleaning, structuring, and transforming the raw data into a consistent and coherent format that can be utilized effectively with a variety of data mining techniques. Essentially, it is about preparing the data so that it becomes a valuable resource for extracting insights, patterns, and knowledge that can drive informed decisions and optimizations within the building’s management and operation.

Specifically, the heterogeneous data sets under consideration encompass a total of 273 sensors, each providing different types of metering data in varying formats, including boolean, real, string, counters, and more. To be more specific, these sensors fall into the following distinct categories: (1) electric energy, (2) heating agent, (3) domestic water, (4) air conditioning, (5) temperature, and (6) humidity meters.

This is why we must extract the data from our IoT architecture to process the data in a way that it is properly filtered and formatted to fit the specifications of the algorithms. In addition to this, data may contain missing values that may alter the accuracy of results provided by the algorithms. Therefore, data should be preprocessed to improve results and obtain more understandable results (see Fig. 2).

In this step, a rough visualization of data collected during the period of time under study can help during the selection process (see for instance Fig. 11). And, depending on the type of analysis the user is interested in, the data can be selected in different periods to perform a short- or long-term analysis.

**Figure 3:**
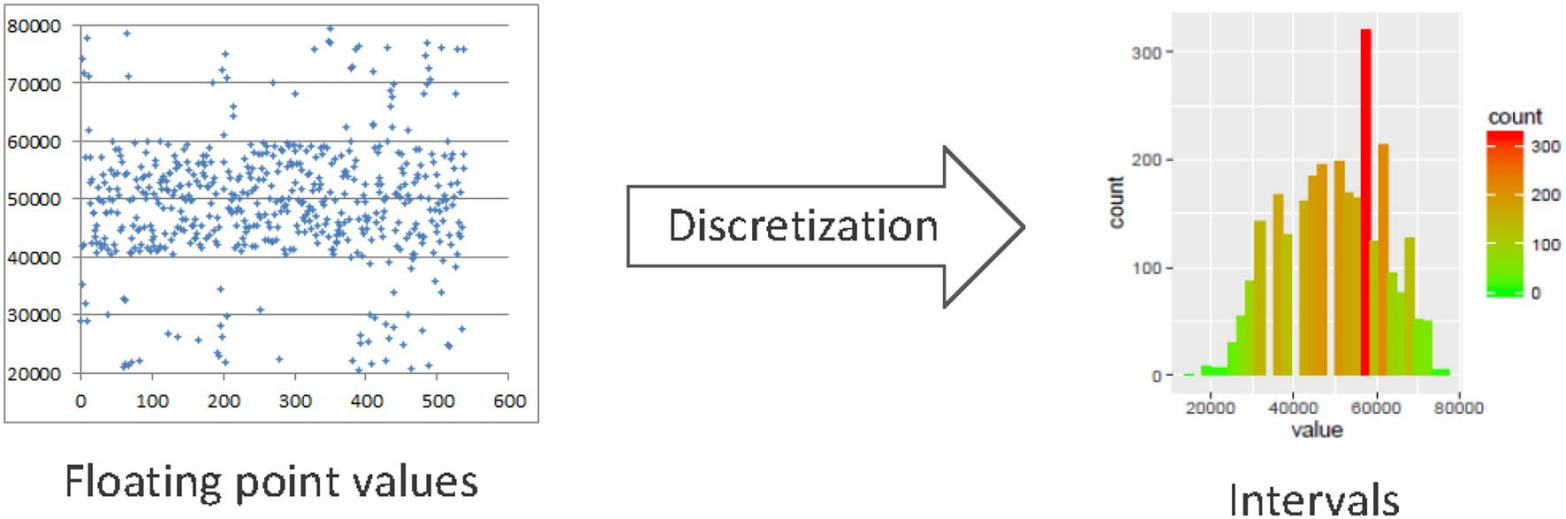
Data discretization process.

In particular for the results presented in this work, we have filtered the available data in order to study the existent associations between the different variables of an office building in different time periods of the year (see Section "Case study" for more details about the periods analysed). Due to the fine granularity of collected data from sensors and metering devices, the data can be aggregated by defining several intervals covering the value ranges of each sensor. This will enable, in a further step of the analysis process, the assignment of linguistic descriptions that may help the user in the final inspection of results. Since we are going to apply association rule mining to the collected data, the aggregation process (also known as the discretization process, see Fig. 3) is very important to obtain more reliable patterns and to reduce the amount of associations obtained. For example, taking into account particular values instead of intervals/linguistic labels the system will produce very similar associations, such as

$$\begin{aligned} \begin{aligned} outdoor\_temp=25^{\circ }C \rightarrow fresh\_air\_handling=20\% \\ outdoor\_temp=24^{\circ }C \rightarrow fresh\_air\_handling=20\% \\ outdoor\_temp=25^{\circ }C \rightarrow fresh\_air\_handling=19\%\\ \end{aligned} \end{aligned}$$

whilst a unique association, such as:

$$\begin{aligned} \begin{aligned} outdoor\_temp&=warm \rightarrow \\ fresh\_air\_handling&=low\_functioning \end{aligned} \end{aligned}$$

or

1 $$\begin{aligned} \begin{aligned} outdoor\_temp&=[22^{\circ }C, 28^{\circ }C] \rightarrow \\ fresh\_air\_handling&=(0\%, 25\%] \end{aligned} \end{aligned}$$

is more descriptive, understandable and summarizes the relationship among a set of values of the variables.

**Figure 4:**
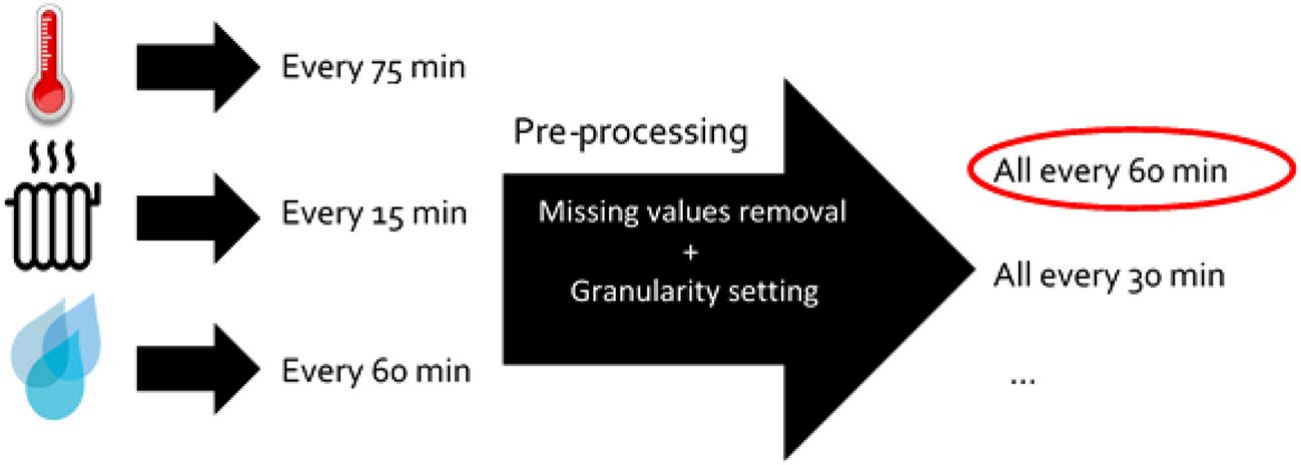
Selecting different granularities for collected data from different types of sensors.

Another problem often found when dealing with sensor data is the different sensor frequency responses. This entails that collected data have different granularity levels because they correspond to values collected in different time instants (see Fig. 4).

**Figure 5:**
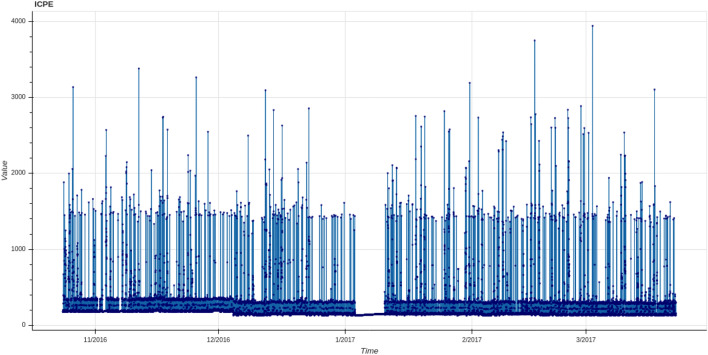
Selecting different granularities for collected data from different types of sensors.

To avoid this, we have selected 60 min as the common granularity level which is available in most sensors of the building. Nevertheless, if necessary, we can select smaller granularity levels by replicating values of those sensors that have higher granularity. In many cases, the low frequency of values given by a sensor happens because its value does not change. Therefore, this justifies the replication of the last value received by the sensor in the time period before next value is received and stored in the database. We can see an example of a change of granularity in the Fig. 5.

Another common problem in building data has to do with consumption sensors. These are usually electricity meters which generate a value that corresponds to the increase in consumption with the last value generated. To explain it better, if the sensor at 10:00 generates a value equal to 100 at 10:15 it could be 125 and at 10:30 138, this increases until it reaches the top of the meter and then it will start again from zero. For the data mining application, we will gather the difference between the values gathered in two-time instants. This is the case of the heat sensor which will entail the consumption of each time interval. Using the previous example, the difference between 10:00 to 10:15 will be 25 and between 10:15 to 10:30, 13.

**Figure 6:**
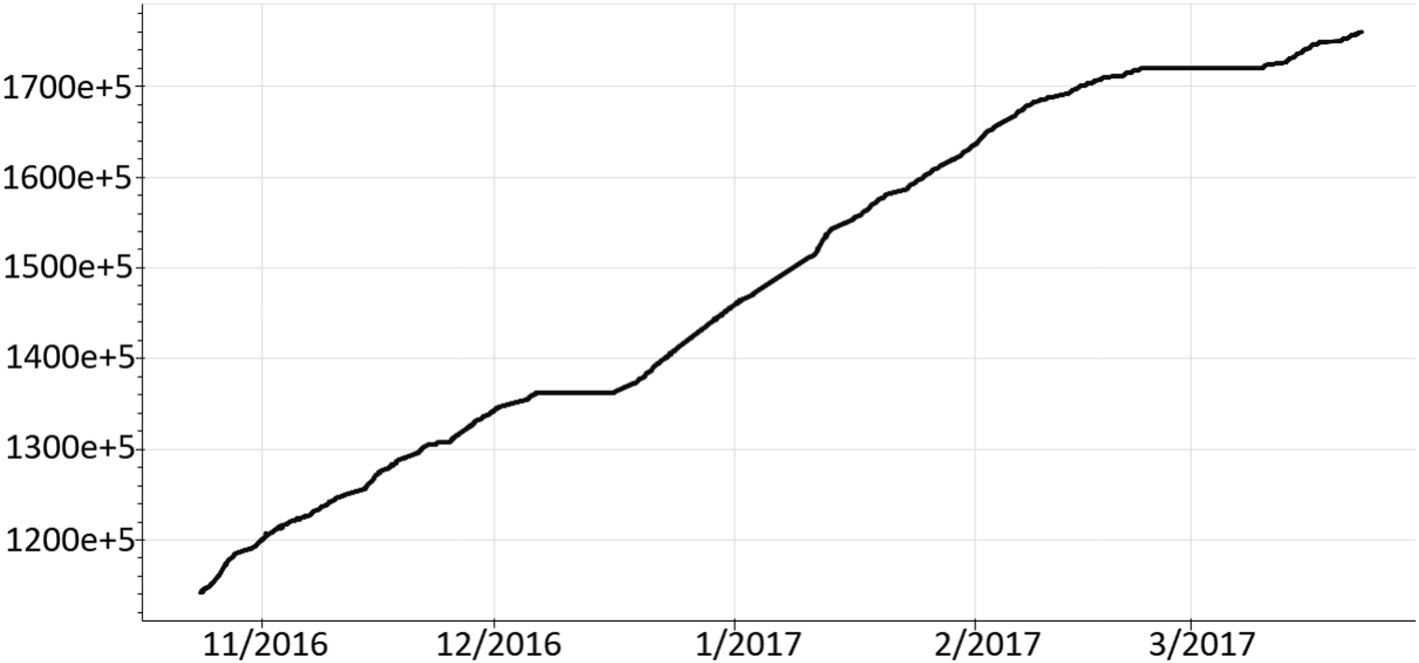
Original values for the consumption variable 9049.

**Figure 7:**
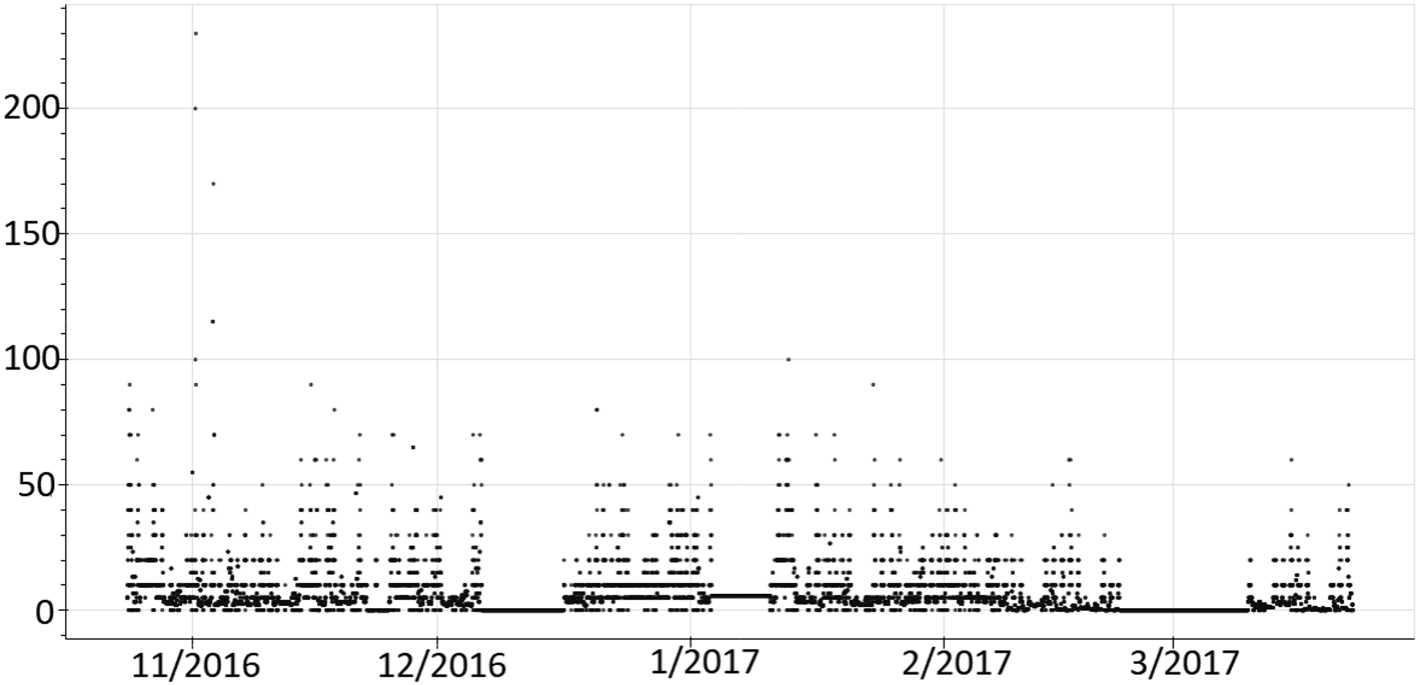
Transformed values for the consumption variable 9049.

This transformation on one of the consumption sensors of the case study can be seen in Fig. 6 where it is represented the original value and Fig. 7 contains the values generated after the transformation.

Additionally, the management of errors in collected data has to be also taken into account. Sensors often fail and, as a consequence, when capturing the measurements given by them they originate some blank cells in the database. To avoid the addition of these missing values to the data that has to be processed by the mining algorithm, we have implemented an automatic removal of missing values when pre-processing the data before storing them in the NoSQL database.

The final stage of the system is to analyse and review the results. This last step is usually carried out by the building operator to find those patterns that contain useful information to find potential energy waste.

### Knowledge discovery method: association rule mining

Association rules depict the frequent co-occurrence of variable values with a high reliability in a database. This is represented by implication rules of the type

$$\begin{aligned} A \rightarrow B \text { support, confidence} \end{aligned}$$

or

$$\begin{aligned} A \rightarrow B \text { support, lift} \end{aligned}$$

where *A*, *B* are values associated to different variables; support is a value in [0, 1] representing the percentage of tuples satisfying *A* and *B* among the total (1 represents all tuples and 0 none), confidence is a value in [0, 1] which indicates how good is the association (1 means totally accurate and 0 means not accurate at all), and lift is a value in the interval $[0,\infty )$ which helps to rank rules according to the ratio between the obtained confidence and the expected confidence. Then, if an association $A\rightarrow B$ is found in a database D, this means that the occurrence of *A* and *B* is frequent and when *A* occurs it tends to occur *B*, measured by the conditional probability of *B* given *A*. *A* is usually called the *antecedent* of the rule, and *B* the *consequent* of the rule. In our scenario each variable corresponds to a unique sensor, device or variable, which presents different values. Then, examples of *A* and *B* can be $sensor_1=value_1$, $sensor_1=value_2$, etc. or for those sensors with discretized values: $sensor=interval_1$, $sensor=interval_2$, $\ldots$ becoming in association rules like the following:

$$\begin{aligned} \begin{aligned} outdoor\_temperature = [18^{\circ }C, 28^{\circ }C]\rightarrow \\ fresh\_air\_handling = (0\%, 25\%], 0.3, 0.8 \end{aligned} \end{aligned}$$

where 0.3 indicates that the 30% of tuples (i.e. sensor measurements) satisfy that the $outdoor\_temparature$ and the $fresh\_air\_handling$ values lie respectively in the intervals $[18^{\circ }C, 28^{\circ }C]$ and $(0\%, 25\%]$; and the 80% of tuples that satisfy the antecedent $(outdoor\_temp.=[18^{\circ }C, 28^{\circ }C])$ also satisfy the consequent $(fresh\_air\_hand.=(0\%, 25\%])$.

**Figure 8:**
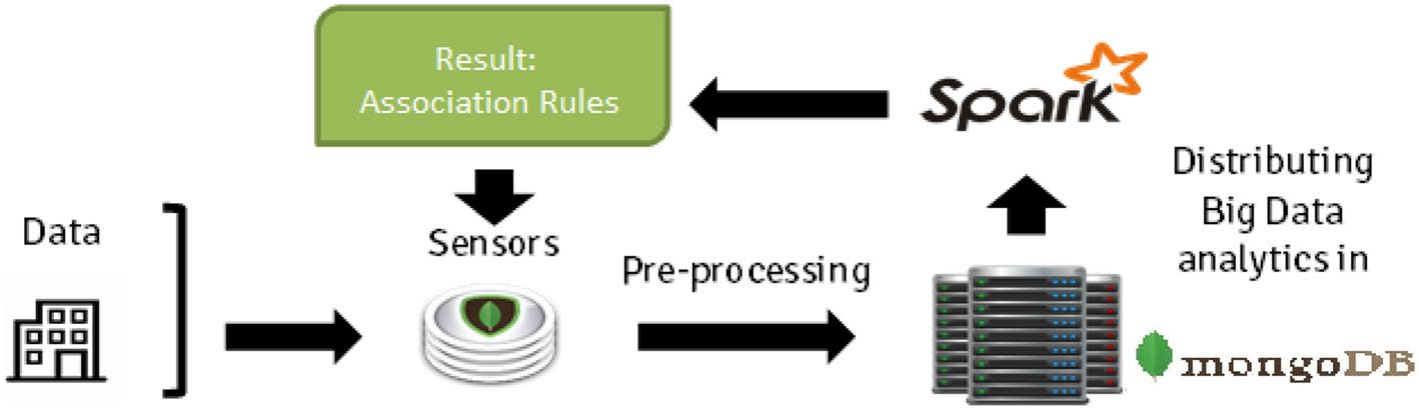
General workflow using the Big Data framework.

However, traditional association rule mining algorithms sometimes fail, usually due to memory constraints, or are very inefficient when they are applied to massive amount of data. This is the case of our scenario, where a completely monitored building usually generates thousands of values per minute. The recently developed framework known as Big Data provides the set of tools for enabling a distributive implementation of traditional mining algorithms in massive datasets. For this reason, we developed the known Apriori and Apriori-TID using the map-reduce framework available in the Apache Spark platform which can deal with databases larger in several magnitude orders than the ones typically managed by classic algorithms. Figure 8 depicts the Big Data analytics workflow followed in the project. The algorithm used in the proposal is the AprioriTID, whose full explanation of the implementation using the Map-Reduce framework can be found at Fernandez-Basso et al.^53,54^.

### Results visualization

The interpretability and understandability of results is essential in every data analysis performed. Although association rule mining produces a comprehensive output in the form of application rules, that are easy to interpret by the experts, in many occasions the volume of obtained rules in some domains can be overwhelming. In these cases, visualization techniques may help to the results inspection in a faster way.

In this work we employed the usual visualization in a table representation plus several different display methods. One of them is a grid representation of rules where at a first glance we can know what are the more frequent and stronger rules (see Figs. 12, 13, 14).

The other method implemented is an interactive visualization which is more flexible and enables to focus in those items the user is more interested to inspect. This type of graph is known as chord diagram, and can be tuned to show all the connections found by the association rule mining algorithm, or to be interactive and only show those relations pointed by the user, using for instance the mouse position (see Figs. 15 and 16).

**Figure 9:**
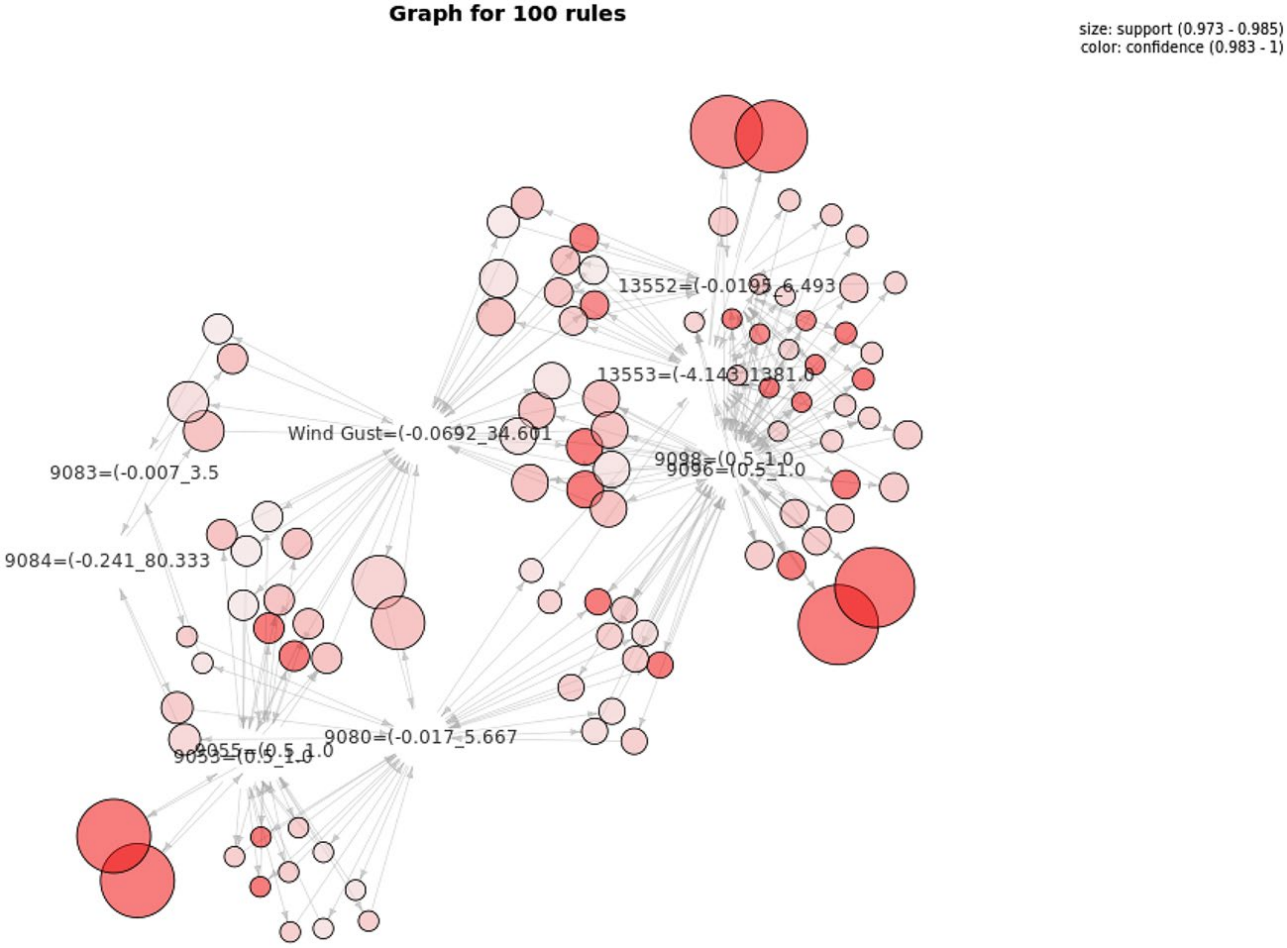
Selection of 100 Association Rules for the winter period.

Graphical visualisations, i.e. graph-based visualizations^55^, were also employed to visualise all the rules and see the interrelationships between them and their attributes. This representation also enables an interactive visualization selecting only a small fraction of rules to be visualized according to different criteria (e.g. exceeding a support or confidence threshold, only involving some specified sensors or devices, etc.). An example is shown in Fig. 9, where 100 rules (out of a total of 18056) have been extracted from the execution of the algorithm with the complete winter dataset.

## Case study

The use case described here was applied in an office building located in Bucharest, Romania. In this building there were different data acquisition elements, sensors of the different components, heating, water, electricity... including the weather forecast and the agendas of the building staff, with which we can extract the occupancy of the building.

The described IoT structure and Data Mining procedure was applied, which allowed the management and collection of the different data sources. From them, we made a selection of data in different time periods to apply the data mining methodology previously described. The aim is to extract patterns of use of the different building’s components in order to improve maintenance, use and performance in different situations, thus improving electricity consumption.

### Ecosystem and system requirements

In this section, we dive into the intricate dynamics within the technology and AI landscape, which intricately shape the operational and design parameters of our energy system. By acquiring a profound understanding of this ecosystem and the exacting technical prerequisites that govern our energy infrastructure, we are equipped to embark on a strategic journey toward a future characterized by the convergence of energy efficiency, resilience, and environmental consciousness.

In our data management system, we employ a comprehensive setup that integrates various components to collect, process, and store data for experimentation. This system is designed to ensure efficiency, scalability, and robustness.

*Data collection* Data collection is orchestrated through a Building Management System (BMS), which acts as the primary data source. The BMS collects data from various sensors and devices within the environment under study.

*Data processing* To handle the substantial volume of data and perform experiments effectively, we have established a powerful computing cluster consisting of three servers, each equipped with 120 CPU cores and 200 GB of RAM. This cluster is configured to work seamlessly with Apache Spark, a distributed data processing framework. Apache Spark enables us to perform data transformation, analysis, and experimentation efficiently across the cluster’s resources.

*Data storage* Our data and experiment results are stored in a NoSQL database, specifically MongoDB. This database system offers flexibility and scalability, crucial for accommodating diverse data types and rapidly evolving experiment requirements.

*High availability and isolation* To ensure data availability and fault tolerance, we deploy two independent MongoDB servers, each containerized using Docker. This configuration improves data redundancy and minimizes the risk of data loss or system downtime. Should one server encounter issues, the other can seamlessly take over to maintain uninterrupted data access.

By implementing this data management system, we can efficiently collect, process, and store data while ensuring the availability and reliability of our experimental infrastructure. This comprehensive approach enables us to conduct experiments with confidence and manage the generated data effectively.

### Description of building, systems and available data

Data collected from an office building in Romania were retrieved for the analysis. The building is located in Bucharest, a city with warm summers and very cold and dry winters. The building is comprised of offices with constant flows of people and fixed-scheduled plans for indoor conditions. Among the rooms we can find several halls, meeting rooms of different sizes, dining rooms and cafeterias.

**Figure 10:**
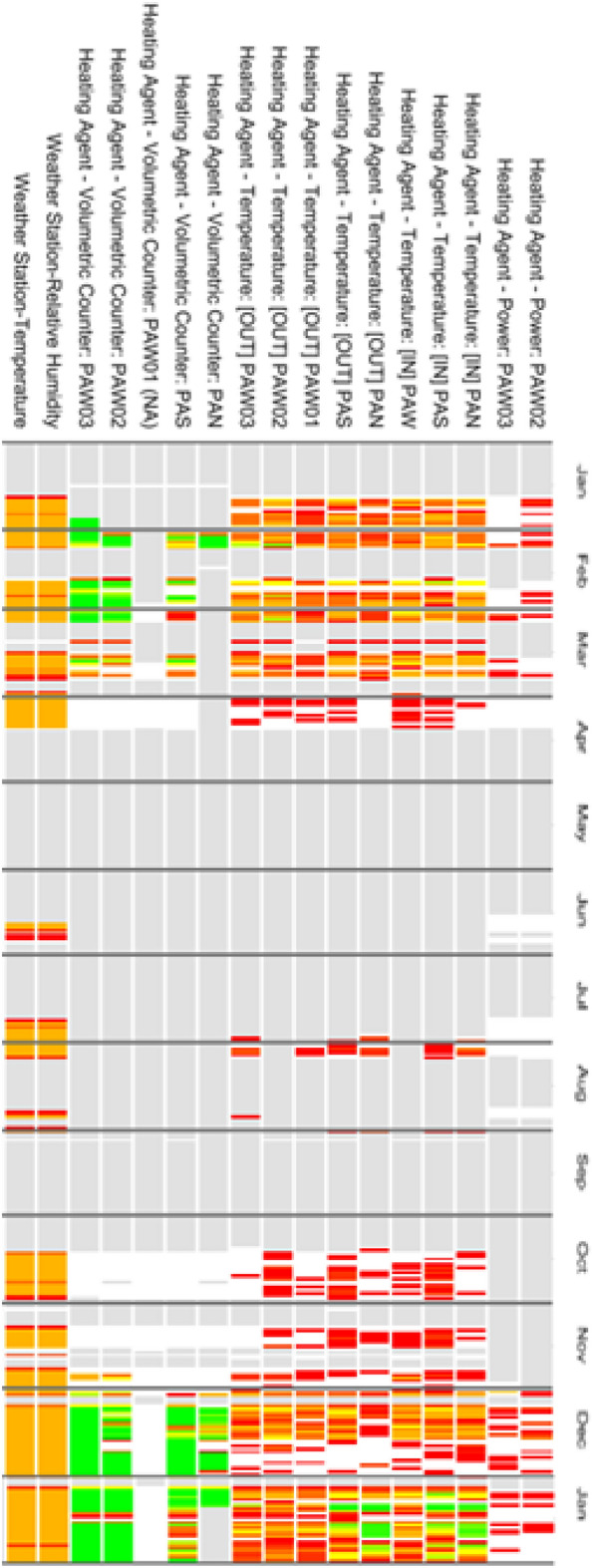
Some of the variables collected in the building during a year period.

The considered set of data comprised 248 sensors corresponding to different metering data that were collected during the first months of 2016 (see Fig. 10 for an example of data collected during a year). The set of sensors can be roughly classified into meters and sensor status. More concretely we distinguish the following groups: (1) electric energy, (2) heating agent, (3) domestic water, (4) air-conditioning, (5) temperature and (6) humidity meters; and (7) setup and status of different sensors (heating, lightning, windows, etc.). Some of these variables refer to their placement in the building. For instance: *North* relates to the north pilot area, and analogously for *South* and *West*.

**Figure 11:**
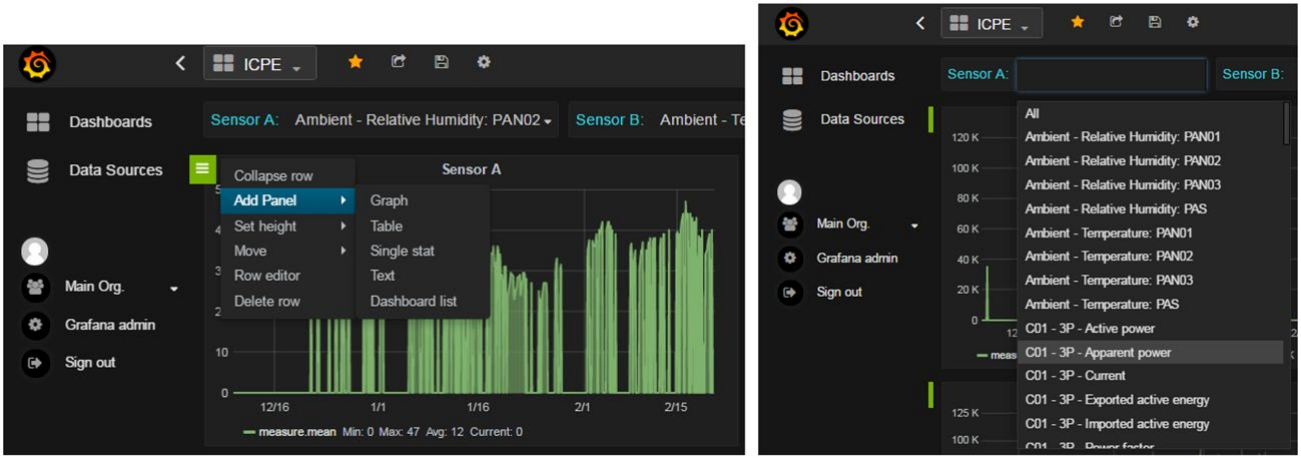
Some screenshots of the data visualization tool.

We have carried out a statistical study of every variable in order to have a representative description of available data. For each variable we have computed its range, frequency and if it contains missing values. To that aim we developed a visualization tool to facilitate data exploration and preprocessing (see Fig. 11 containing some screenshots of the tool integrated in IoT environment).

We conducted different experiments in three periods of time: 1 month, 3 months and 10 days: (A) from 1st January 2016 to 31st March 2016, (B) the whole month of January 2016, and C) a shorter period that comprises data from 4th to 14th January 2016. These periods have been chosen to test the performance of the methodology in shorter and bigger datasets.

We have delimited the range of variables to a selection of 34 variables, which were pointed out as important by the building’s manager. These variables have been pre-processed following the process explained in Section "Data selection and preprocessing from IoT architecture", becoming in a total of 57 items when the discretization of real-valued sensors was made. Specifically, among the collected variables in this scenario, we can find a wide variety of value types including integer, Boolean, character strings and real values. The latter type was discretized in three different intervals according to the variables distribution:

$$\begin{aligned} \begin{aligned} \left[ min, \frac{max-min}{3}\right) ,\\ \left[ \frac{max-min}{3},\frac{2(max-min)}{3} \right) ,\\ \left[ \frac{2(max-min)}{3}, max \right] , \\ \end{aligned} \end{aligned}$$

where *min* and *max* represent the minimum and maximum values of the variable respectively.

### Identifying typical building behavioural patterns

The results described here were obtained by imposing the minimum support threshold as 5% and minimum confidence as 0.6 (60% of accuracy), indicating that at least the 5% of transactions must satisfy the items contained in the extracted rule and the rule must have a confidence value higher or equal to 0.6. These values used are due to the fact that, being sensors, the support of many of them is very low and therefore it is necessary to reduce this value to 0.05 in order to extract a set of frequent itemsets in order to extract enough frequent itemsets to find interesting rules. Different sets of results were obtained for the different tests carried out. The number of results obtained was over 800 when only one week was used and over 10 000 when the whole dataset was used.

**Figure 12:**
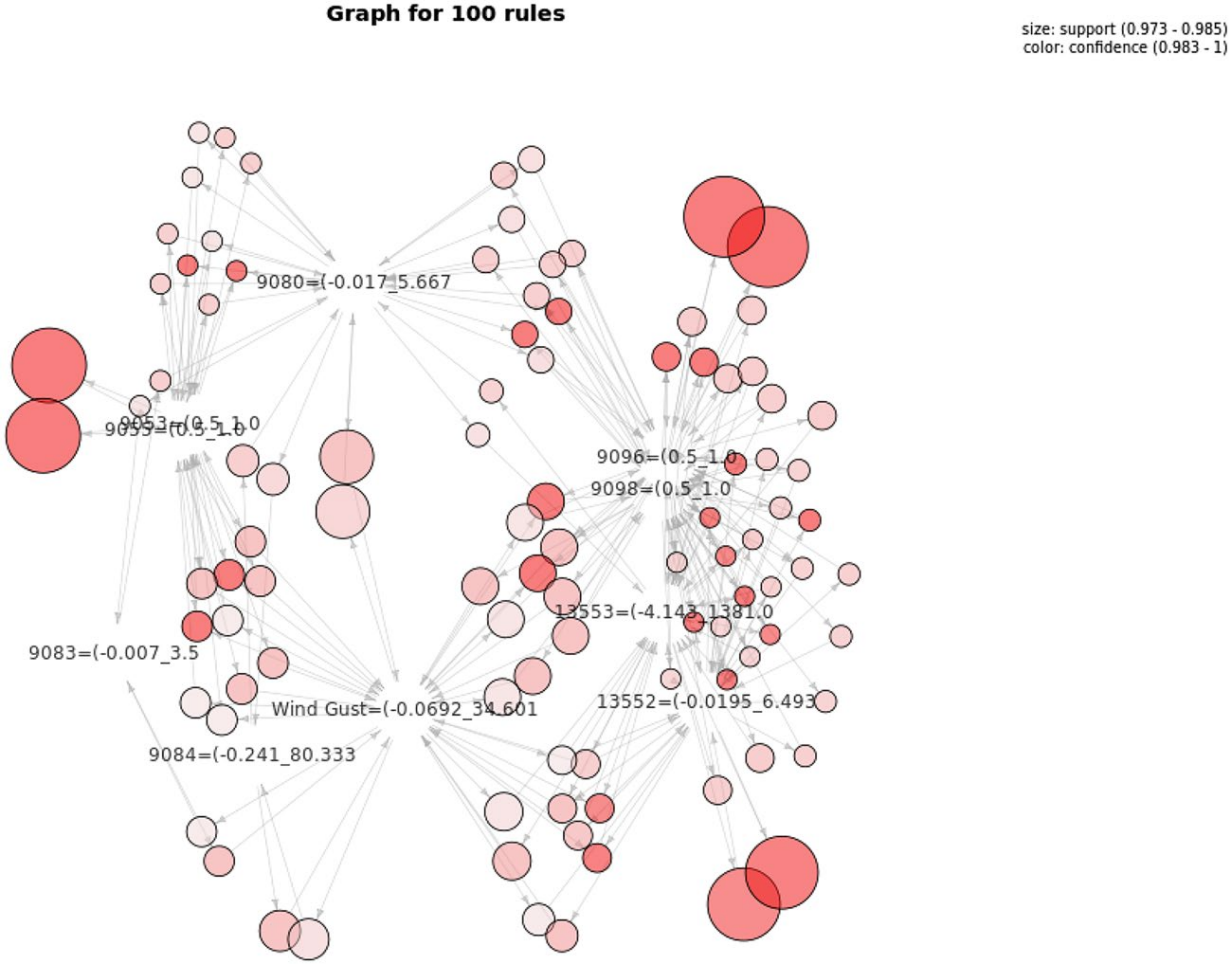
Association Rules for the period: 4th–14th January(using sensor keys to improve visualisation).

**Figure 13:**
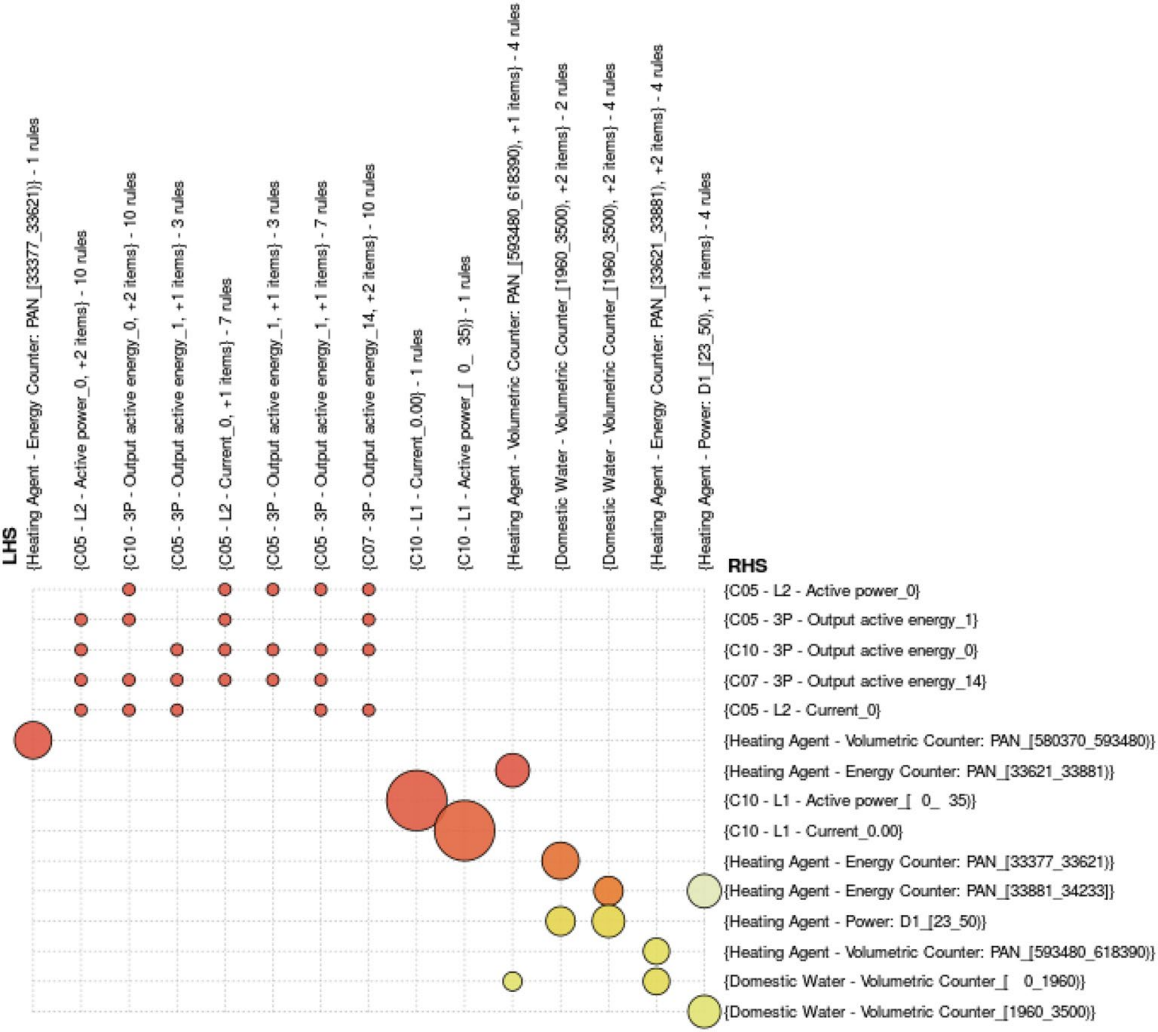
Association Rules for the period: 1st–31st January.

**Figure 14:**
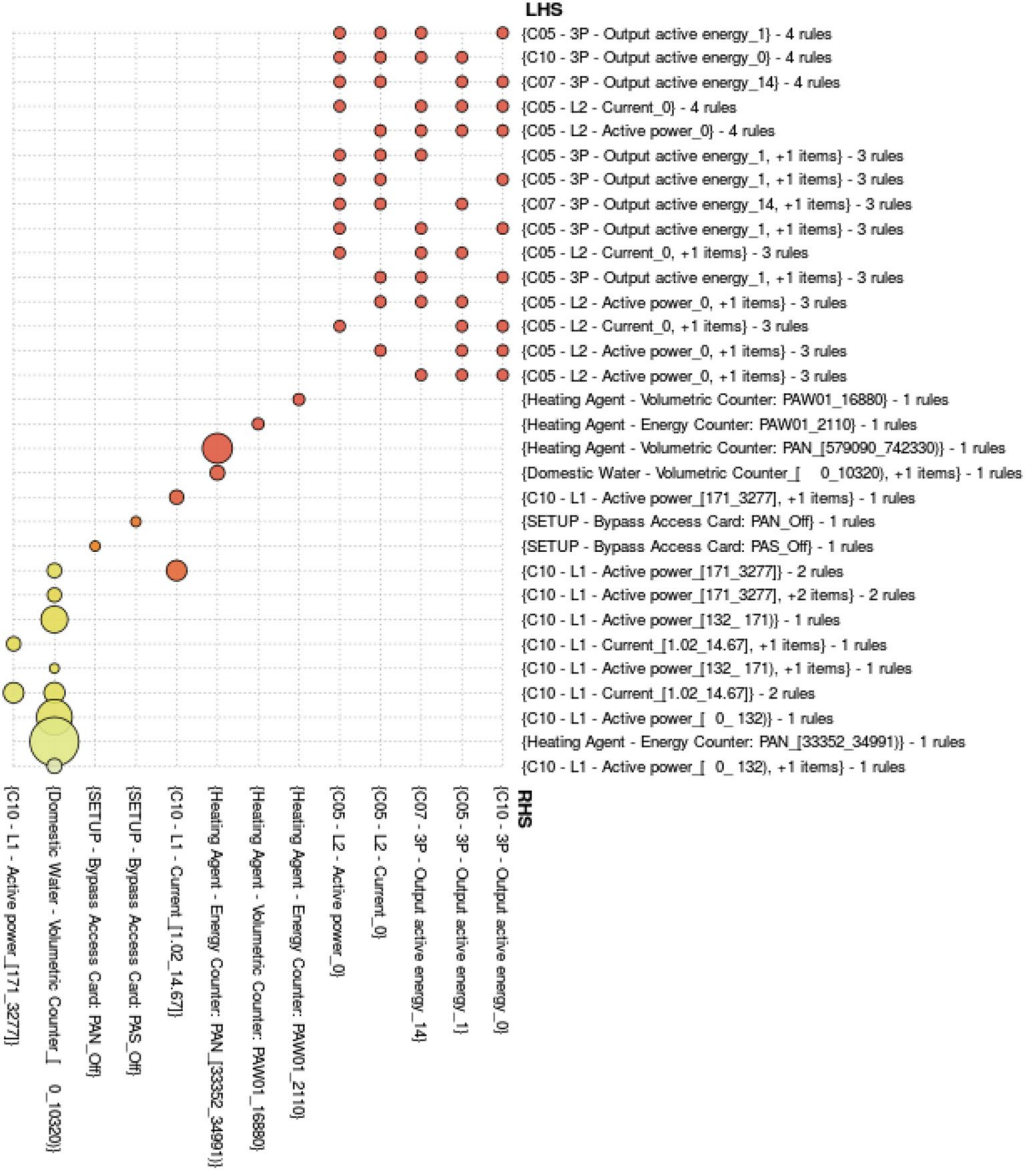
Association Rules for the period: 1st January–31st March.

In order to facilitate the visualization of extracted associations we have developed a visualization tool for association rules that enables different types of visualization by means of an intermediate representation of rules in JSON. Particularly, the visualization used in Figs. 12, 13, 14 enables, at a first glance, which are the associations with higher support and confidence values. Particularly, in them we can see the results obtained for the three selected time periods. In these figures, the higher the size of circles, the higher the support value of the rule, and the darker the colour are, the higher the confidence value is. Concretely in Figs. 13 and 14, orange colour represents confidence values near to 1 (very confident associations), and lighter yellow confidence values near to the minimum confidence 0.6 (less confident associations). Due to space restrictions, the graphs only show those rules with support higher to 0.1. The horizontal axis contains the antecedent (LHS—Left Hand Side) of the association rules and the vertical axis the consequent (RHS—Right Hand Side) of the rules.

In Fig. 13, we can observe some direct relations between power sensors (current and active power) that do not provide meaningful information, since they are directly related. However some interesting rules relate the heating with the domestic water and some energy consumption values which justify the use of central heating in winter months to heat the water above its initial temperature. This tendency can be also seen in Figs. 13 and 14 with higher frequency but lower confidence (lighter yellow).

Some of the discovered rules through the inspection of Figs. 12, 13, 14 relates electricity consumption by the associated single phase measurements in certain areas of the building. To highlight one example:

$$\begin{aligned} \texttt { (Room10: Current [1.01,14.67),} \texttt {North: Heating [33352,34018))}\\ \longrightarrow \texttt {Room10: Power [162,3277)} \end{aligned}$$

with support of 0.058 and high confidence of 0.965. This type of rule indicates a relationship among different ranges of measurements taken by the sensors. In this case, the obtained relation does not reveal abnormal situations in the functioning of the electricity and the heating.

**Figure 15:**
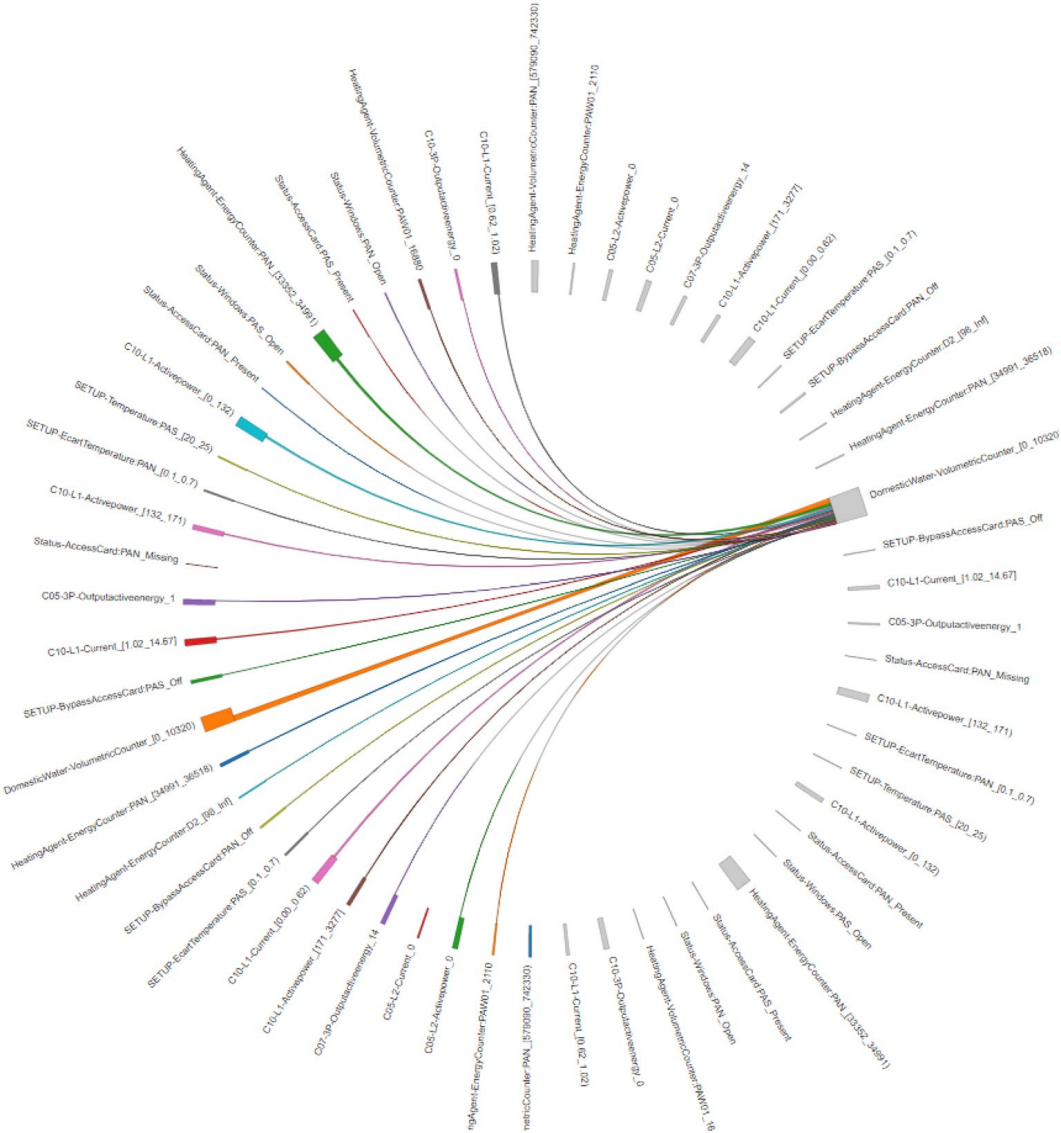
Association rules involving domestic water meter for the period 1st–31st January.

**Figure 16:**
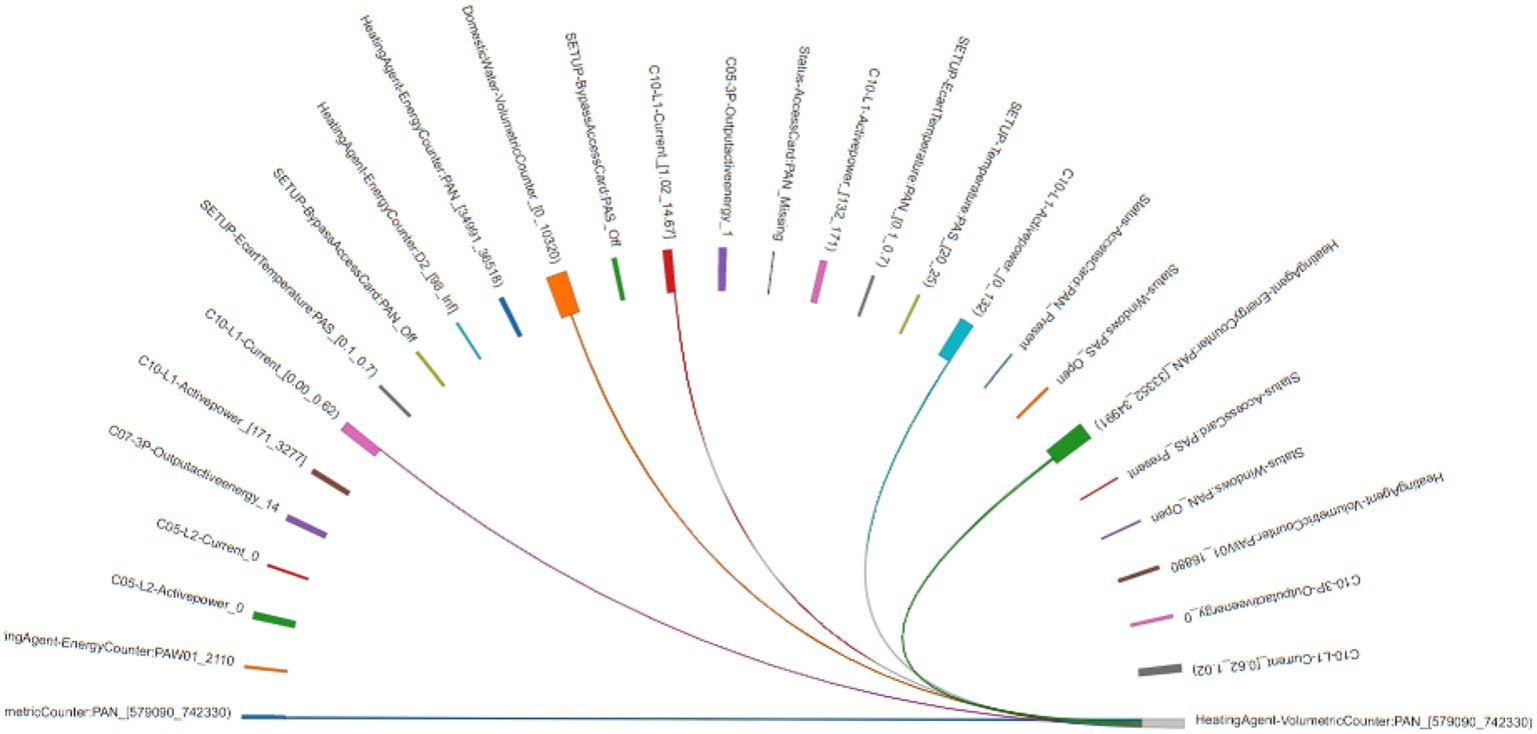
Association rules involving heating volume counter for the period 1st–31st January.

A different graphical interface was employed to navigate through the different selected variables and see their connections with other items (Figs. 15 and 16). The main advantage of the chord diagrams used in these figures is that they are interactive, so they enable a deeper inspection focusing in each of the variables. In Fig. 15, we have analysed the volume of consumed water to see how it is associated with other sensors. The size of the rectangles adjacent to the variables indicates the strength of the association (i.e. the confidence value). However, it is not easy to know, at the same time, both measures (support and confidence) using this type of visualization. It is important to note that the variables are repeated in both hemispheres of the circular representation, indicating their presence in the antecedent (south hemisphere) or in the consequent (north hemisphere). This can be observed in Fig. 16 where only an hemisphere was considered to show the associations with the heating counter values.

In Fig. 15 we can observe that the Domestic water meter, which is a general sensor measuring the total consumption of water in the building, is related to many other types of variables. This happened because this variable is very frequent in the whole database. Therefore, we have to be careful with this kind of items that may offer many spurious relations, making difficult the discovery of useful associations.

Additionally, through the inspection of the whole set of association rules in the three periods, we can highlight several association rules. The first type of rule found in the whole month of January relates to the absence of people in some areas of the building with no consumption of electricity. This was due that several rooms were almost always empty in that period. An example of this type of rule is:

$$\begin{aligned}{} & {} \texttt {(Room10:Current 0),(Room10:Power 0)} \rightarrow \\{} & {} \texttt {(North:Bypass Access-OFF)} \end{aligned}$$

with support around and lift around.

Additionally, an interesting rule also appeared:

$$\begin{aligned}{} & {} \texttt {(South:Access Card-Present)} \rightarrow \\{} & {} \texttt {(South:Windows-Open)} \end{aligned}$$

with support around 0.05 and confidence around 0.6, indicating a direct relation of the presence of people working in the south area offices and the opening of windows in that area, usually for air circulation purposes. This could mean that the climate control of the south area does not work adequately and the comfort is lost. By delimiting the time that this occur, the building manager can analyse it and prevent an unnecessary waste of energy.

The obtained rules can be used to identify behavioural patterns of the energy behaviour of the building, but also about the interaction of users with some parts of the building, like this last rule indicates. The absence of analogous rules in other areas of the building could indicate that only in the south area there is a necessity of improving air circulation mechanisms in order to avoid opening the window for achieving a lower waste of energy in heating consumption.

The proposed system allows to obtain knowledge from the general functioning of the building. This will also include the processing of information coming from additional sensors that might be installed in the building like for instance sensors for earthquakes, fires, floods, etc. The system enables the use of this information in order to detect possible anomalies or emergencies.

## Conclusions and future improvements

In this work, a Data Mining methodology has been implemented using the Big Data framework and applied to different sets of data collected from an office building in Romania. All this methodological development has been applied following an IoT architecture. This architecture has allowed the management and storage of heterogeneous data sets to later apply Big Data mining algorithms to analyse them. In particular, we have applied an association rule mining algorithm which has been developed using the Spark platform in order to being able of handling the amount of data generated by sensors in the building. This technique has enabled the exploitation of sensor data collected in different pilot areas of a building.

The proposed solution has been applied to data collected over three different time periods, revealing various relationships that illuminate the energy behaviour of the building. These findings have revealed distinct patterns which, as discussed, can be used to improve the energy efficiency of the building. In conclusion, this paper has demonstrated the effectiveness of using Big data tools, specifically association rules, to simplify the tasks of building managers. Through this system, building managers can easily identify and address a number of critical issues, including (1) potential sensor malfunctions, (2) unexplored energy saving opportunities, (3) inefficient configuration settings that contribute to energy waste, and even (4) user behaviour that results in energy waste. This innovative approach provides a comprehensive solution for improving building energy efficiency and overall management.

This system has coexisted with control systems^1,56^ in the Energy IN TIME project in a parallel way. The knowledge extraction system helps the end-user to know how the building works thanks to historical data. This allows getting a deeper and more extensive knowledge of how to design a future control system.

A limitation of the system arises when automatically analysing continuously generated data should be handled. This leads us to propose a future improvement of the proposed mining techniques for handling with such continuous flow and process it in real time conveniently. To do so, there are recently developed utilities within the Spark framework that enables processing stream data called Spark Streaming^57,58^. This extension will enable to process live data streams by dividing them into batches which can be then processed by the Spark’s mining algorithms.

Regarding the possible applications of the presented methodology, the system was deployed as a pilot in Faro’s airport. The aim was to adapt the boarding and arrivals rooms to the number of passengers waiting for the aircraft based on the flight time and the aircraft occupancy. In general, the system can be applied to sensorized spaces where the increase of users’ comfort is pursued.

## Data Availability

The data supporting the conclusions of this study are available from the ICPE building. However, restrictions apply to the availability of the ICPE BMS dataset, which is not publicly available. For more information on the data and the results of the study please contact Carlos Fernandez-Basso (cjferba@decsai.ugr.es carlos.basso@ucl.ac.uk).
